# Altered serum acylcarnitine profile is associated with the status of nonalcoholic fatty liver disease (NAFLD) and NAFLD-related hepatocellular carcinoma

**DOI:** 10.1038/s41598-019-47216-2

**Published:** 2019-07-23

**Authors:** Kenichiro Enooku, Hayato Nakagawa, Naoto Fujiwara, Mayuko Kondo, Tatsuya Minami, Yujin Hoshida, Junji Shibahara, Ryosuke Tateishi, Kazuhiko Koike

**Affiliations:** 1 Department of Gastroenterology, The University of Tokyo, 7-3-1 Hongo, Bunkyo-ku, Tokyo, 113-8655 Japan; 2 Liver Tumor Translational Research Program, Simmons Comprehensive Cancer Center, Division of Digestive and Liver Diseases, Department of Internal Medicine, University of Texas Southwestern Medical Center, Dallas, TX 75390 USA; 3 Department of Pathology, The University of Tokyo, 7-3-1 Hongo, Bunkyo-ku, Tokyo, 113-8655 Japan

**Keywords:** Non-alcoholic fatty liver disease, Non-alcoholic steatohepatitis

## Abstract

Metabolic disturbance of lipids is a hallmark of nonalcoholic fatty liver disease (NAFLD). In this study, we measured the serum levels of 15 acylcarnitine species of various carbon chain lengths from 2 to 18 in 241 patients with biopsy-proven NAFLD, including 23 patients with hepatocellular carcinoma (HCC), and analyzed the relationship between serum acylcarnitine profile and NAFLD status. Long-chain acylcarnitines AC14:1 and AC18:1 increased gradually with the progression of fibrosis and further increased in patients with HCC, whereas the middle-chain acylcarnitine AC5:0 exhibited the opposite trend. In particular, AC18:1, which we previously showed to possess a tumor promoting effect, was significantly elevated in patients with HCC compared to those without HCC. In addition, long-chain acylcarntines including AC18:1 were positively correlated with serum levels of inflammatory cytokines. Although none of the acylcarnitine species were independently associated with the presence of HCC, (AC16:0 + AC18:1)/AC2:0, an index for the diagnosis of carnitine palmitoyltransferase 2 (CPT2) deficiency, was independently associated with the presence of HCC after adjusting for age and liver fibrosis stage, likely reflecting the downregulation of CPT2 in HCC tissues. Thus, serum acylcarnitine profiles changed significantly according to the status of NAFLD, which may be implicated in the pathogenesis of NAFLD.

## Introduction

Nonalcoholic fatty liver disease (NAFLD) is the most common chronic liver disease, affecting 25% of the general population worldwide^1,2^. Nonalcoholic steatohepatitis (NASH) is considered the progressive form of NAFLD and is characterized by liver steatosis, inflammation, hepatocellular injury, and fibrosis that eventually results in cirrhosis and/or hepatocellular carcinoma (HCC)^3^. NASH is currently the second leading cause of liver transplantation in the USA and is estimated to become the leading indication for liver transplantation in the near future^4^.

NAFLD is characterized by the accumulation of various lipid species within hepatocytes. Hepatic *de novo* lipogenesis is paradoxically enhanced despite increased influx of lipids to the liver from insulin-resistant adipose tissue and dietary fat, which results in a marked accumulation of lipids in hepatocytes^5,6^. In addition, hepatic fatty acid β-oxidation (FAO) is impaired in some patients with NAFLD despite increased lipid storage^7^. Such disturbed lipid metabolism causes a marked accumulation of toxic lipids in hepatocytes, leading to lipotoxic cell death, inflammation, subsequent fibrosis, and HCC^8,9^.

We recently identified acylcarnitine species as metabolites that accumulate specifically in obesity- and NASH-related HCC tissues using untargeted metabolomics profiling in mouse HCC models^10^. Acylcarnitine is the intermediate metabolite produced during FAO. Fatty acids are converted to acyl-coenzyme A (acyl-CoA) and then to acylcarnitine via conjugation to carnitine by carnitine palmitoyltransferase (CPT) 1A. After mitochondrial translocation, CPT2 converts acylcarnitine to free carnitine and acyl-CoA, which subsequently enters the FAO pathway. The accumulation of acylcarnitine in obesity- and NASH-related HCC tissues has been attributed to downregulation of CPT2, which is also observed in human NAFLD-related HCC tissues. In addition, patients with NAFLD and HCC had increased serum levels of acylcarnitine. Interestingly, serum levels of acylcarnitine increased gradually with the progression of fibrosis in patients with NAFLD and further increased in patients with NAFLD and HCC, whereas no such relationship was observed for free carnitine. Notably, several recent studies have indicated the possible effects of acylcarnitine species on disparate aspects of pathophysiology, such as inflammation and insulin resistance^11–13^. We also reported that oleoylcarnitine (AC18:1), one of the long-chain acylcarnitine species, functions as an oncometabolite by conferring stem cell properties to HCC cells through activation of signal transducer and activator of transcription 3^10^. Therefore, altered composition of acylcarnitine species may contribute to the pathophysiology of NAFLD. However, little is known about the relationships between serum acylcarnitine profiles and the status of NAFLD.

In this study, we measured serum levels of 15 acylcarnitine species of various carbon chain lengths from AC2 to AC18, including unsaturated acylcarnitines, using liquid chromatography-tandem mass spectrometry (LC-MS/MS) in a large cohort of 241 biopsy-proven NAFLD patients with and without HCC, and analyzed the relationships between their serum acylcarnitine profiles and NAFLD status.

## Results

### Patient characteristics

A total of 241 biopsy-proven NAFLD patients, including 23 patients with HCC, were enrolled in the study. Their demographic characteristics are listed in Table 1. Free carnitine (C0), acetylcarnitine (AC2:0), propionylcarnitine (AC3:0), butyrylcarnitine (AC4:0), isovalerylcarnitine (AC5:0), hydroxyisovalerylcarnitine (AC5-OH), glutarylcarnitine (AC5-DC), hexanoylcarnitine (AC6:0), octanoylcarnitine (AC8:0), decanoylcarnitine (AC10:0), dodecanoylcarnitine (AC12:0), myristoylcarnitine (AC14:0), tetradecanoylcarnitine (AC14:1), palmitoylcarnitine (AC16:0), octadecanoylcarnitine (AC18:0), and oleoylcarnitine (AC18:1) were measured by LC-MS/MS. As AC5-OH, AC5-DC, and AC14:0 were undetectable in the majority of patients with NAFLD in this cohort (96.7%, 91.3%, and 98.3%, respectively), the values of these acylcarnitines were omitted from Table 1. Serum levels of free carnitine were similar between patients with and without HCC, whereas serum levels of total acylcarnitine were significantly higher in patients with HCC than in those without HCC. Among acylcarnitine species, only AC18:1 was significantly higher in patients with HCC than in those without HCC. In contrast, AC5:0 was significantly lower in patients with HCC than in those without HCC.

**Table 1:** Baseline characteristics of patients with NAFLD with or without HCC.

Variable	Over all (n = 241)	HCC (n = 23)	Non-HCC (n = 218)	*p* value
Gender, male, n (%)	135 (56)	14 (61)	121 (56)	0.66
Age (year)	58 (45–67)	72 (65–76)	55 (44–65)	<0.001
BMI (kg/m^2^)	28.3 (25.5–31.2)	28.0 (25.4–29.6)	28.3 (25.6–31.2)	0.73
Platelet count (×10^3^/μL)	211 (166–244)	126 (83–182)	215 (173–250)	<0.001
Albumin (g/dL)	4.0 (3.7–4.2)	3.4 (3.2–4.0)	4.0 (3.8–4.2)	<0.001
AST (IU/L)	39 (28–60)	35 (27–39)	40 (28–62)	0.14
ALT (IU/L)	53 (33–86)	25 (19–33)	57 (37–91)	<0.001
Fibrosis (0/1/2/3/4), n (%)	38/74/41/63/25 (16/31/17/26/10)	1/0/2/10/10 (4/0/9/43/43)	37/74/39/53/15 (17/34/18/24/7)	<0.001
Steatosis (1/2/3), n (%)	133/73/35 (55/30/15)	21/1/2 (91/4/4)	112/72/34 (51/33/16)	<0.001
Lobular inflammation (0/1/2/3), n (%)	16/152/67/6 (7/63/28/2)	1/18/4/0 (4/78/17/0)	15/134/63/6 (7/61/29/3)	0.56
Hepatocyte ballooning (0/1/2), n (%)	74/117/50 (31/49/20)	6/13/4 (26/57/17)	68/104/46 (31/48/21)	0.8
Total Acylcarnitine (enzymatic cycling method) (μmol/L)	9.7 (8.0–11.5)	10.2 (9.5–13.2)	9.6 (7.9–11.2)	0.01
Free carnitine (enzymatic cycling method) (μmol/L)	53.7 (48.7–58.3)	56.5 (44.7–59.0)	53.4 (48.7–58.2)	0.69
**Acylcarnitine species** (**LC-MS/MS**)				
C0 (μmol/L)	50.6 (42.8–55.9)	51 (40.3–60.7)	50.6 (43.1–55.8)	0.86
AC2:0 (μmol/L)	7.31 (5.98–9.23)	7.16 (6.24–8.83)	7.36 (5.93–9.26)	0.94
AC3:0 (μmol/L)	0.423 (0.355–0.524)	0.466 (0.371–0.578)	0.422 (0.352–0.52)	0.38
AC4:0 (μmol/L)	0.153 (0.124–0.188)	0.149 (0.113–0.178)	0.153 (0.126–0.188)	0.42
AC5:0 (μmol/L)	0.072 (0–0.091)	0.06 (0–0.075)	0.073 (0–0.092)	0.03
AC6:0 (μmol/L)	0 (0–0.05)	0 (0–0.063)	0 (0–0.056)	0.45
AC8:0 (μmol/L)	0.089 (0.066–0.138)	0.09 (0.079–0.116)	0.08 (0.065–0.139)	0.38
AC10:0 (μmol/L)	0.134 (0.093–0.219)	0.134 (0.115–0.172)	0.134 (0.09–0.219)	0.48
AC12:0 (μmol/L)	0 (0–0.064)	0 (0–0.063)	0 (0–0.064)	0.98
AC14:1 (μmol/L)	0.062 (0.085–0.116)	0.097 (0.081–0.131)	0.085 (0.015–0.11)	0.08
AC16:0 (μmol/L)	0.114 (0.098–0.136)	0.114 (0.103–0.134)	0.114 (0.098–0.135)	0.64
AC18:0 (μmol/L)	0.042 (0.035–0.048)	0.038 (0.034–0.046)	0.042 (0.035–0.048)	0.4
AC18:1 (μmol/L)	0.661 (0.554–0.807)	0.73 (0.645–0.87)	0.649 (0.543–0.805)	0.03
**Tumor status**				
Tumor size (cm)		2.1 (1.8–2.8)		
Number of nodules (Solitary/2 or 3 nodules), n (%)		16/7 (70/30)		

Data are expressed as median (25^th^–75^th^ percentiles) unless otherwise indicated. HCC, hepatocellular carcinoma; BMI, body mass index; AST, aspartate aminotransferase; ALT, alanine transaminase.

### Serum levels of acylcarnitine species in patients with NAFLD

**Figure 1:**
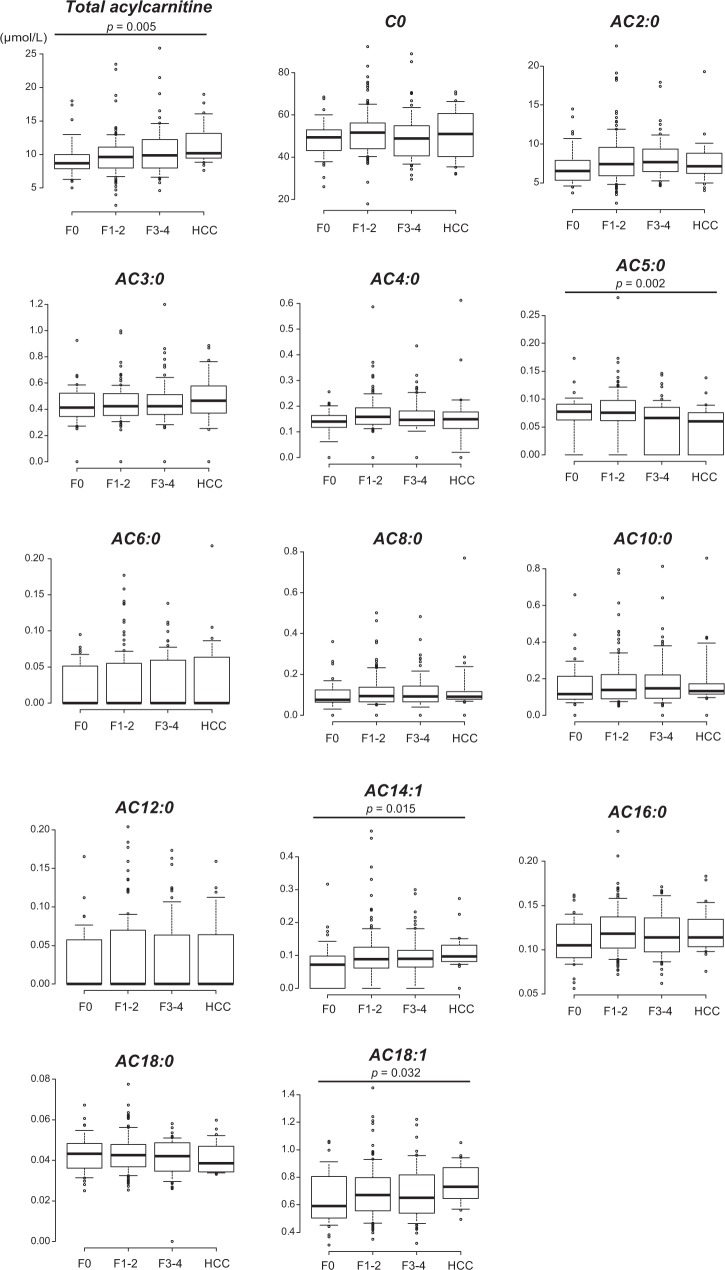
Serum levels of acylcarnitine species in nonalcoholic fatty liver disease (NAFLD) patients with and without hepatocellular carcinoma (HCC). Patients without HCC were classified according to their fibrosis stage. The box plot indicates the mean (horizontal line), interquartile range (box), 10^th^ and 90^th^ percentiles (whiskers), and outliers outside the 10^th^ and 90^th^ percentiles (dots).

**Figure 2:**
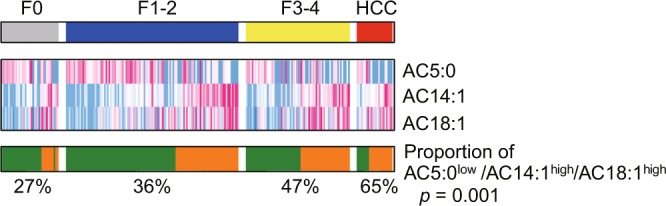
The proportion of patients with the AC5:0^low^/AC14:1^high^/AC18:1^high^ signature according to NAFLD status.

We previously reported that serum levels of total acylcarnitine increased according to the progression of fibrosis, and further increased after HCC development in patients with NAFLD^10^; therefore, we analyzed the associations between serum levels of acylcarnitine species and liver fibrosis stage and HCC in this cohort (Fig. 1). Consistent with our previous study, serum levels of total acylcarnitine increased gradually with the progression of fibrosis and further increased in patients with HCC in this cohort. Among acylcarnitine species, AC14:1 and AC18:1 also revealed increasing trends. In contrast, serum levels of AC5:0 decreased gradually with the progression of fibrosis and further decreased in patients with HCC. Based on these findings, we created a signature for predicting advanced stages of NAFLD using the nearest template prediction method, and the proportion of patients who had a signature of AC5:0^low^/AC14:1^high^/AC18:1^high^ increased significantly with NAFLD disease progression (*p* = 0.001 by Cochran–Armitage test) (Fig. 2).

We also analyzed the relationships between serum levels of acylcarnitine species and liver steatosis, inflammation, and hepatocyte ballooning, but no significant correlations were detected (Supplementary Figs 1–3). Only AC5:0 was positively correlated with liver steatosis and negatively correlated with hepatocyte ballooning, both of which were of borderline significance (*p* = 0.056 and 0.088, respectively).

Since the kidney also plays an important role in carnitine homeostasis through filtration and tubular reabsorption of carnitine and acylcarnitines^14,15^, we analyzed the relationships between serum levels of acylcarnitine species and estimated glomerular filtration rate (eGFR) in this cohort. Most of short- and medium-chain acylcarnitines (AC2:0, AC3:0, AC4:0, AC5:0, AC8:0, and AC10:0) in addition to C0 and total acylcarnitine were significantly negatively correlated with eGFR, while no significant correlations were detected between long-chain acylcarnitines and eGFR (Supplementary Table 1).

### Relationships between serum levels of acylcarnitine species and inflammatory mediators

Acylcarnitines, particularly medium- and long-chain acylcarnitines, have been reported to activate proinflammatory signaling pathways in monocytes and induce the secretion of inflammatory cytokines and chemokines, including tumor necrosis factor α (TNF-α), interleukin-6 (IL-6), and monocyte chemoattractant protein 1 (MCP-1)^11,16,17^, all of which have been suggested to be involved in the pathogenesis and progression of NASH. Therefore, we evaluated the relationships between serum levels of acylcarnitine species and these inflammatory mediators in 199 NAFLD patients from this cohort (Table 2). Long-chain acylcarnitines, AC16:0, AC18:0, and AC18:1, were significantly correlated with serum TNF-α levels. Medium-chain acylcarnitines (AC6:0, AC8:0, AC10:0, and AC12:0) and long-chain acylcarnitines (AC14:1 and AC18:1) were positively correlated with IL-6, whereas AC5:0 was negatively correlated with IL-6. None of acylcarnitine species were significantly correlated with serum levels of MCP-1, although AC14:1 and AC16:0 had weak correlations with borderline significance.

**Table 2:** Correlations between acylcarnitine species and inflammatory mediators.

Acylcarnitine species	TNFα		IL-6		MCP1	
	Spearman’s Rho	*p* value	Spearman’s Rho	*p* value	Spearman’s Rho	*p* value
C0	0.05	0.48	0.03	0.68	0.04	0.61
AC2:0	0.07	0.31	0.10	0.15	0.05	0.49
AC3:0	0.04	0.55	0.03	0.71	0.09	0.22
AC4:0	0.11	0.14	−0.01	0.91	0.10	0.18
AC5:0	0.04	0.56	−0.16	0.02	0.00	0.95
AC6:0	0.02	0.78	0.14	0.04	0.07	0.34
AC8:0	−0.01	0.90	0.19	0.01	0.06	0.41
AC10:0	−0.02	0.82	0.21	0.003	0.05	0.48
AC12:0	−0.05	0.47	0.19	0.01	0.09	0.20
AC14:1	0.05	0.46	0.27	<0.001	0.13	0.08
AC16:0	0.25	<0.001	0.13	0.06	0.12	0.09
AC18:0	0.17	0.02	−0.03	0.68	0.06	0.43
AC18:1	0.24	<0.001	0.20	0.004	0.11	0.12

### (AC16:0 + AC18:1)/AC2:0 as a diagnostic index of CPT2 deficiency is associated with HCC in NAFLD patients

**Figure 3:**
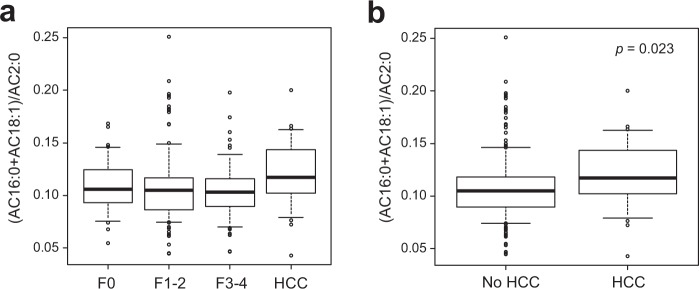
The values of the (AC16:0 + AC18:1)/AC2:0 index in NAFLD patients with or without HCC. Patients without HCC were classified according to their fibrosis stage (**a**) and non-classified (**b**). The box plot indicates the mean (horizontal line), interquartile range (box), 10^th^ and 90^th^ percentiles (whiskers), and outliers outside the 10^th^ and 90^th^ percentiles (dots).

Next, we explored aylcarnitine species that were independently associated with the presence of HCC in NAFLD patients. Although serum levels of AC18:1 and AC5:0 were significantly altered in NAFLD patients with HCC (Table 1), the progression of liver fibrosis also affected the serum levels of AC18:1 and AC5:0 (Fig. 1), and thus serum AC18:1 and AC5:0 were not independent indicators for the presence of HCC after adjusting for liver fibrosis stage. We previously showed that the accumulation of acylcarnitine in NAFLD-related HCC tissues was caused by downregulation of CPT2, whereas the expression of CPT2 in non-tumorous tissues was not altered by liver fibrosis^10^. Based on these findings, we adopted the index (AC16:0 + AC18:1)/AC2:0 to diagnose congenital deficiency of CPT2^18^ as an indicator for the presence of HCC in NAFLD patients. As shown in Fig. 3a,b, although the (AC16:0 + AC18:1)/AC2:0 index was not affected by the degree of liver fibrosis, the index was significantly higher in NAFLD patients with HCC compared to those without HCC. Furthermore, the (AC16:0 + AC18:1)/AC2:0 index was an independent factor associated with the presence of HCC after adjusting for age and liver fibrosis stage (Table 3).

**Table 3:** Factors associated with HCC in patients with NAFLD.

Variable	Univariate analyses		Multivariate analyses	
	Odds ratio	*p* value	Odds ratio	*p* value
Age (per 1 year)	1.17 (1.09–1.25)	<0.001	1.16 (1.07–1.25)	<0.001
Gender, male	1.25 (0.51–3.00)	0.62		
BMI (per 1 kg/m^2^)	0.99 (0.9–1.09)	0.91		
Fibrosis stage (per 1 stage)	3.48 (2.05–5.91)	<0.001	2.87 (1.50–5.51)	0.002
AC5:0 (per 0.01 μmol/L)	0.89 (0.81–1.00)	0.03		
AC18:1 (per 0.1 μmol/L)	1.19 (0.96–1.47)	0.1		
(AC16:0 + AC18:1)/AC2:0 (per 0.01)	1.12 (1.00–1.27)	0.05	1.25 (1.05–1.48)	0.01

## Discussion

In the present study, serum acylcarnitine profiles were associated with various aspects of the pathophysiology of NAFLD. Especially, AC18:1 was significantly positively correlated with serum levels of inflammatory cytokines and significantly increased in NAFLD patients with HCC, which were consistent with our previous mouse experiments^10^. To date, several studies have reported associations between acylcarnitine profiles and liver disease^19–22^. However, those studies included relatively small numbers of NAFLD patients, and, to the best of our knowledge, this is the largest study to assess the relationships between acylcarnitine species and the status of NAFLD.

Serum levels of unsaturated long-chain acylcarnitine species, such as AC14:1 and AC18:1 increased with the progression of liver fibrosis and HCC, whereas medium-chain acylcarnitine AC5:0 showed the opposite trend in our cohort. As a result, the proportion of patients who had the AC5:0^low^/AC14:1^high^/AC18:1^high^ signature increased significantly with the progression of NAFLD. In contrast, serum acylcarnitine profiles did not affect liver steatosis, inflammation, or hepatocyte ballooning. Pérez-Carreras *et al*. reported an increase in long-chain acylcarnitine and a decrease in short-chain acylcarnitine in the serum of patients with NASH compared to control subjects, and Peng *et al*. reported that only AC18:1 increased in the serum of patients with NASH compared to obese control subjects^7,20^. These findings are largely consistent with our results. However, as these studies contained small numbers of patients with NASH (43 and 32 patients, respectively), no detailed analysis of the relationship between the pathological findings of NAFLD and the serum acylcarnitine profiles was performed. Increased long-chain acylcarnitine and decreased short- and middle-chain acylcarnitine were observed in patients with chronic hepatitis B virus (HBV) infection^19^, consistent with our results. Therefore, such changes in serum acylcarnitine profiles may be commonly caused by the progression of liver fibrosis, although further studies are needed.

We also found that (AC16:0 + AC18:1)/AC2:0, a diagnostic index for congenital deficiency of CPT2, was independently associated with HCC in patients with NAFLD. Because AC2:0 is an end product of mitochondrial FAO, a high (AC16:0 + AC18:1)/AC2:0 index reflects increased generation and decreased degradation of acylcarnitine. In patients with NAFLD, a large amount of fatty acids is delivered to the liver as materials of acylcarnitine, whereas FAO is frequently suppressed in HCC tissue due to downregulation of CPT2, which may result in a higher index value. Consistent with our results, Lu *et al*. recently reported that the serum and tissue levels of long-chain acylcarnitine species increased but those of short- and middle-chain acylcarnitine species decreased in patients with hepatitis virus-related HCC, and such changes in acylcarnitine profiles were caused by downregulation of CPT2^22^. In contrast, Zhou *et al*. reported that serum acylcarnitine profiles were similar between patients with advanced fibrosis and HCC in those with chronic HBV infection^19^. Such differences among studies may be attributed to the degree of CPT2 downregulation in HCC. As we previously reported^10^, downregulation of CPT2 is more frequently seen in steatohepatitic HCC that often occurs in patients with steatohepatitis with or without hepatitis virus infection^23,24^. Peroxisome proliferator-activated receptor α (PPARα) mainly regulates the expression of CPT2, and a recent study showed that activation of β-catenin plays a critical role in the upregulation of PPARα and CPT2 in HCC^25^. Importantly, the frequency of mutation and activation of β-catenin is significantly low in steatohepatitic HCC^26,27^. Therefore, the (AC16:0 + AC18:1)/AC2:0 index may be more useful for predicting HCC in patients with steatohepatitis and could be a surrogate marker for activation states of β-catenin and FAO in HCC. Furthermore, AC18:1, which we previously showed possesses a tumor promoting effect in mouse models^10^, increased significantly in NAFLD patients with HCC, suggesting that AC18:1 may act as an oncometabolite in human hepatocarcinogenesis.

Various acylcarnitine species were associated with serum levels of inflammatory cytokines in patients with NAFLD. TNF-α and IL-6 play pivotal roles in NAFLD progression and HCC development in mouse models^28–31^. Acylcarnitines, particularly medium- and long-chain acylcarnitines, reportedly induce the secretion of inflammatory cytokines through activation of the Toll-like receptor/MyD88 signaling-mediated nuclear factor κB pathway^11,16,17^. Therefore, acylcarnitine released from hepatocytes may also be involved in the pathogenesis of NAFLD by stimulating the secretion of inflammatory cytokines from immune cells.

We also analyzed the relationships between serum levels of acylcarnitine species and renal function in NAFLD patients and found that short- and medium-chain acylcarnitines, but not long-chain acylcarnitines, were significantly negatively correlated with eGFR. Although the mechanism is unclear, these findings were consistent with a previous study analyzing the patients with diabetic kidney disease^32^. In other words, long-chain acylcarnitines can reflect the status of NAFLD without being affected by renal function.

This study had several limitations. First, data on the amounts of acylcarnitine species in the liver and HCC tissues were not available. As acylcarnitine species can also be released from other tissues, the changes in serum acylcarnitine profiles may not directly reflect the status of acylcarnitine metabolism and FAO in the liver and HCC tissues. In particular, skeletal muscle is another major source of acylcarnitines, but we did not have data on skeletal muscle volume in our NAFLD cohort. Since sarcopenia is associated with poor outcomes in patients with liver diseases^33^, the relationships between serum acylcarnitine profiles and the skeletal muscle volume need to be explored. Second, although carnitine homeostasis is maintained by dietary intake (mainly meat products) as well as endogenous synthesis and renal reabsorption^34^, we did not have data on dietary habits in this cohort. Third, although we and another group previously identified downregulation of CPT2 as a critical factor for acylcarnitine accumulation in HCC tissues^10,22^, the mechanism underlying the changes in serum acylcarnitine profiles during the progression of NAFLD remains unclear. One possibility is that FAO in patients with advanced-stage NASH may be impaired in the process of acyl-CoA dehydrogenation that is catalyzed by acyl-CoA dehydrogenases including very long-chain acyl-CoA dehydrogenase (VLCAD), long-chain acyl-CoA dehydrogenase (LCAD), and medium-chain acyl-CoA dehydrogenase (MCAD). In fact, patients with VLCAD deficiency show significant elevations of serum long-chain acylcarnitine species^35^. Since the expressions and functions of acyl-CoA dehydrogenases in patients with NAFLD are poorly understood, further studies are needed.

In summary, serum acylcarnitine profiles changed significantly with the progression of NAFLD. The roles of acylcarnitines in the pathogenesis of NAFLD merit further investigation.

## Methods

### Patients

Levels of acylcarnitine species were analyzed in serum samples collected from 241 patients with biopsy-proven NAFLD with (n = 23) and without HCC (n = 218) at the University of Tokyo Hospital from November 2011 to December 2017. The recruitment criteria of liver biopsy for patients without HCC were as follows: liver transient elastography measured by FibroScan >7.0 kPa; persistent elevations of serum aspartate aminotransferase and alanine aminotransferase above the upper limit for at least 6 months; fatty liver diagnosis based on ultrasound examination showing an increase in hepatorenal contrast, history of alcohol consumption <30 g/day for males or <20 g/day for females; seronegativity for hepatitis B virus surface antigen and hepatitis C virus antibody; and absence of autoimmune hepatitis, primary biliary cholangitis, primary sclerosing cholangitis, Budd–Chiari syndrome, Wilson disease, and drug-induced liver injury. All patients with HCC were treated with radiofrequency ablation and simultaneously underwent biopsies of non-tumor liver tissues to diagnose underlying liver diseases. All serum samples were obtained in the morning on the day of the liver biopsy and/or radiofrequency ablation after an overnight fast. Serum samples were frozen at −70 °C until assay. Among the 241 serum samples, 180, including 18 samples obtained from patients with HCC, were the same samples as used in our previous study analyzing serum total acylcarnitine levels^10^, and 61 samples, including five samples obtained from patients with HCC, were newly added in this study. None of the patients included in this study were supplemented with L-carnitine. eGFR was calculated using the following formula established by the working group of the Japanese Chronic Kidney Disease Initiative: eGFR (mL/min/1.73 m^2^) = 194 × (serum creatinine)^−1.094^ × (age)^−0.287^ (×0.739 for women)^36^. The study protocol conformed to the ethical guidelines of the Declaration of Helsinki and was approved by the Ethics Committee of the University of Tokyo. All patients provided informed consent.

### Serum concentrations of acylcarnitine species

The serum concentrations of acylcarnitines were analyzed by LC-MS/MS at Sekisui Medical Co., Ltd (Tokyo, Japan). The NeoSMAAT AC kit, a new reagent composed of 15 types of acylcarnitine species, was used to create the calibration curves to measure the serum concentrations of acylcarnitine species more accurately.

### Serum concentrations of total acylcarnitine, free carnitine, and inflammatory mediators

Serum levels of total acylcarnitine and free carnitine were measured using the enzymatic cycling method at SRL Inc. (Tokyo, Japan). Serum levels of TNF-α, IL-6, and MCP-1 were also measured at SRL Inc. Among the 241 serum samples, 42 were not subjected to analyses of inflammatory mediators due to a limited sample volume.

### Histopathological assessment

Liver biopsy samples were obtained from the right lobe using a 16 G needle with a 20-mm long biopsy specimen notch. All samples were assessed by experienced pathologists blinded to the clinical data and the study design. Liver histology was assessed according to the criteria of the Nonalcoholic Steatohepatitis Clinical Research Network^37^.

### Statistical analyses

Intergroup differences of continuous and categorical variables were tested using the Mann–Whitney *U*-test and Fisher’s exact test, respectively. The increasing and decreasing tendencies of acylcarnitine species across the categorical data were assessed using the Jonckheere–Terpstra trend test. Serum acylcarnitine signature-based prediction of advanced NAFLD was determined using the nearest template prediction algorithm^38^. In this analysis, patients were classified into the two groups based on the “cosine distance” from the hypothetical template. If the distance is greater than 0, the patient was recognized as high. Correlations between serum levels of acylcarnitine species and continuous variables were analyzed using Spearman’s rank correlation coefficient analysis. Univariate and multivariate analyses were performed for the presence of HCC using a logistic regression model with a stepwise procedure. Variables with *P*-values < 0.1 were included in the multivariate analysis. A two-tailed *p* < 0.05 was considered significant. All data analyses were performed using the *R* statistical software (www.r-project.org).

## Supplementary information

Supplementary information

## Data Availability

The datasets generated during and/or analyzed during the current study are available from the corresponding author on reasonable request.
