# Supplementary material for: Altered serum acylcarnitine profile is associated with the status of nonalcoholic fatty liver disease (NAFLD) and NAFLD-related hepatocellular carcinoma
Source: Sci Rep. 2019 Jul 23;9:10663. doi: 10.1038/s41598-019-47216-2 (PMC6650415; doi:10.1038/s41598-019-47216-2)
Supplement: Supplementary file 1 — Supplementary information [file 41598_2019_47216_MOESM1_ESM.pdf]

Altered serum acylcarnitine profile is associated with the status of nonalcoholic fatty liver disease (NAFLD) and NAFLD-related hepatocellular carcinoma

Kenichiro Enooku, Hayato Nakagawa, Naoto Fujiwara, Mayuko Kondo, Tatsuya Minami, Yujin Hoshida, Junji Shibahara, Ryosuke Tateishi, Kazuhiko Koike

Supplementary Figure legends

Supplementary Figure 1. Relationships between serum levels of acylcarnitine species and liver steatosis grade in patients with NAFLD.

Supplementary Figure 2. Relationships between serum levels of acylcarnitine species and lobular inflammation grade in patients with NAFLD.

Supplementary Figure 3. Relationships between serum levels of acylcarnitine species and hepatocyte ballooning grade in patients with NAFLD.

Supplementary figure 1

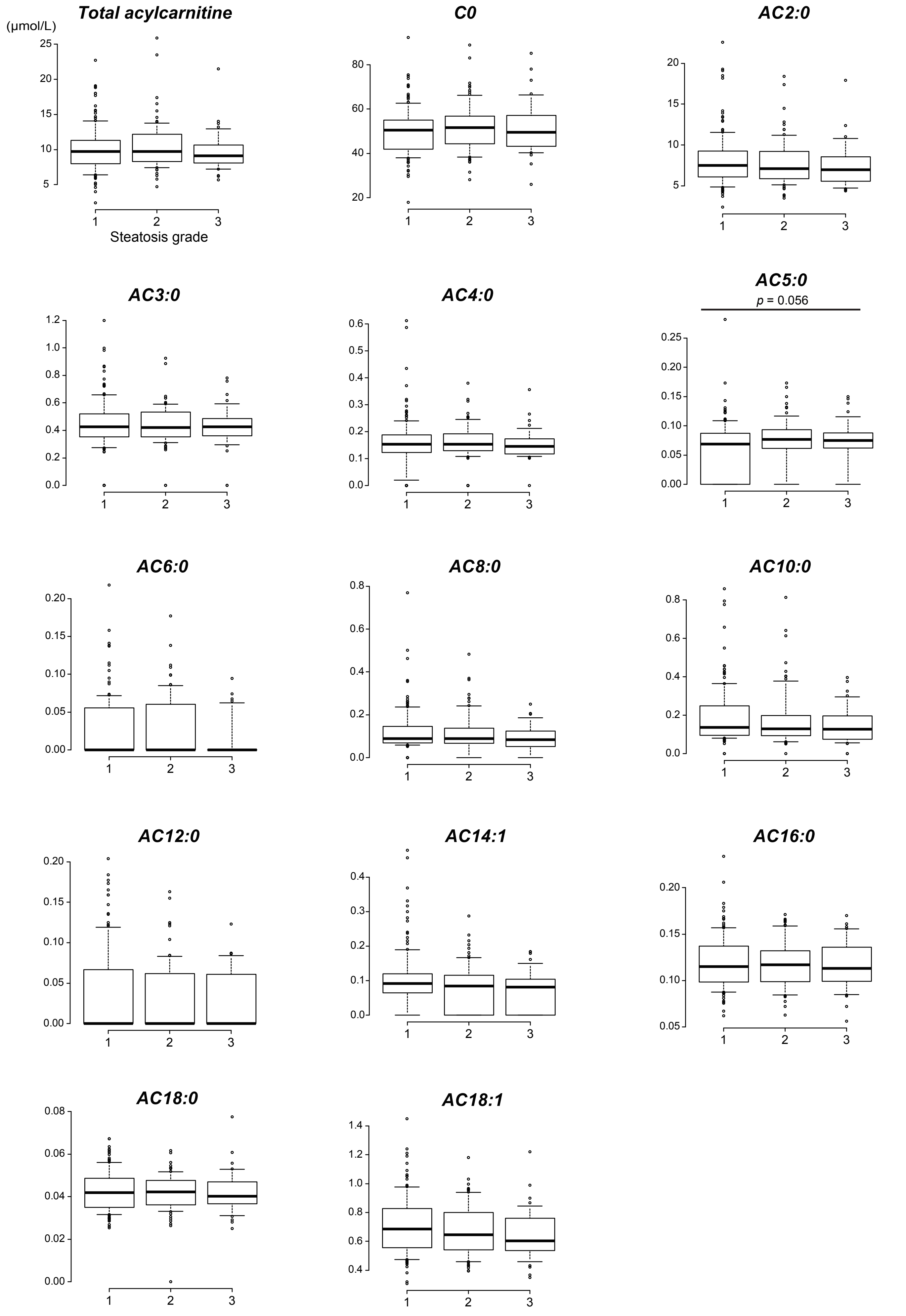

Supplementary figure 2

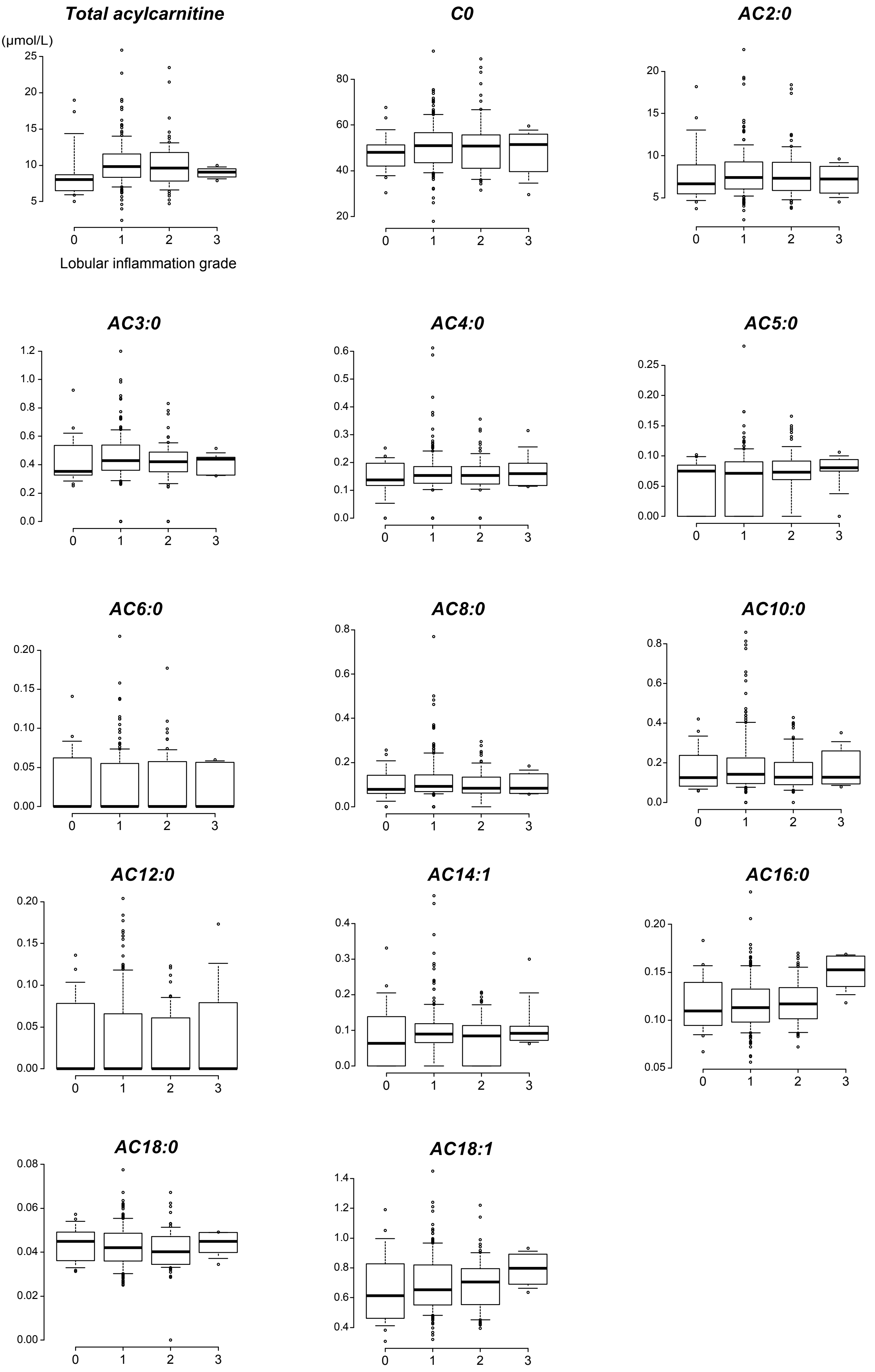

Supplementary figure 3

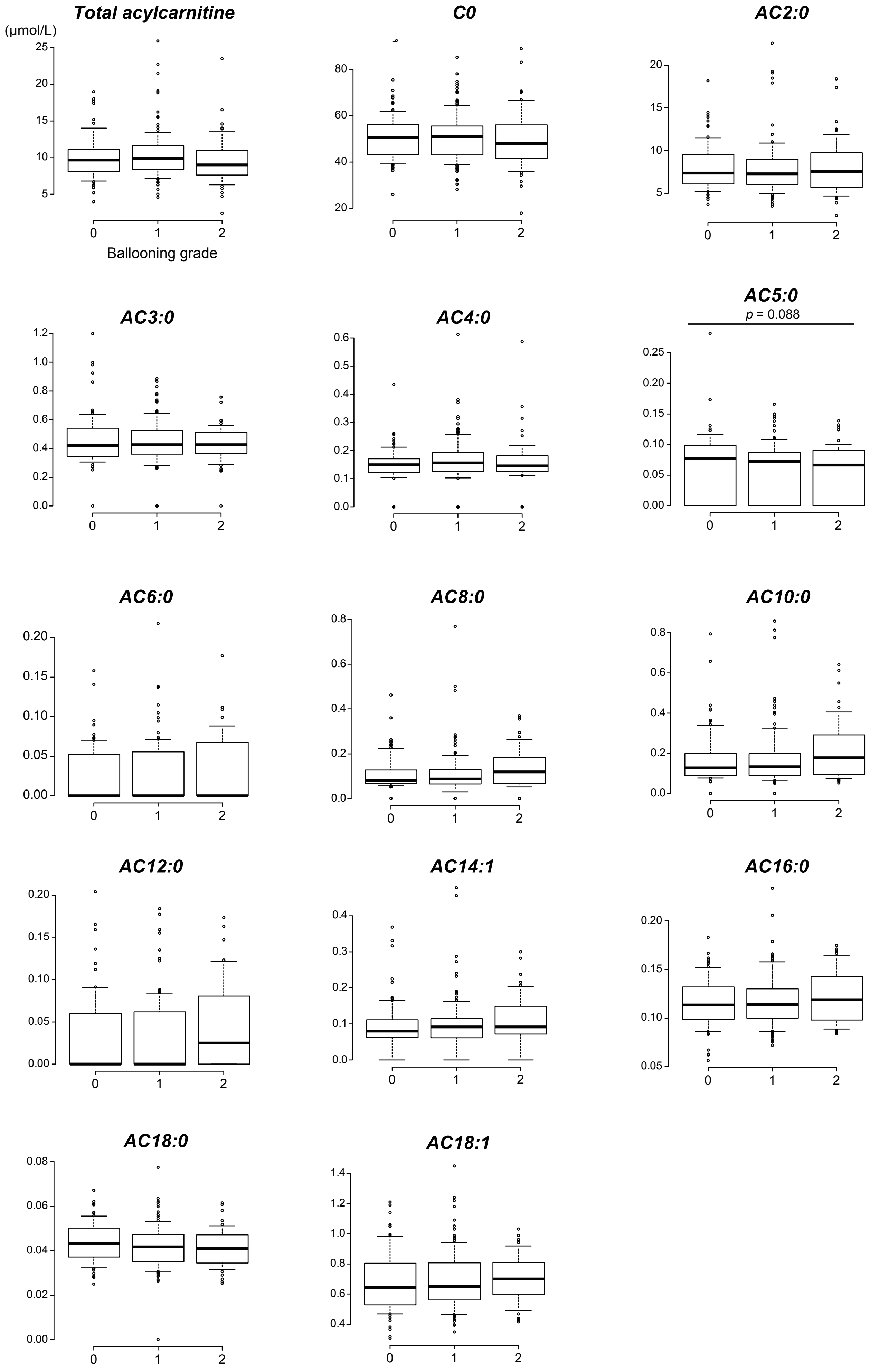

Supplementary table 1. Correlations between acylcarnitine species and estimated glomerular filtration rate

	eGFR	
Acylcarnitine species	Spearman's Rho	*p* value
Total acylcarnitine	-0.17	0.008
C0	-0.21	0.001
AC2:0	-0.16	0.01
AC3:0	-0.17	0.008
AC4:0	-0.29	< 0.001
AC5:0	-0.14	0.02
AC6:0	0.06	0.31
AC8:0	-0.16	0.01
AC10:0	-0.16	0.01
AC12:0	-0.01	0.93
AC14:1	-0.09	0.16
AC16:0	-0.04	0.51
AC18:0	-0.04	0.59
AC18:1	-0.05	0.43
